# State of the practice of health information systems: a survey study amongst health care professionals in intellectual disability care

**DOI:** 10.1186/s12913-021-07256-9

**Published:** 2021-11-18

**Authors:** Joep Tummers, Hilde Tobi, Bianca Schalk, Bedir Tekinerdogan, Geraline Leusink

**Affiliations:** 1 Information Technology, Wageningen University & Research, Hollandseweg 1, 6701KN, Wageningen, The Netherlands; 2 Department of Primary and Community Care, Radboud University Medical Center, P.O. Box 9101, 6500 HB Nijmegen, The Netherlands; 3 Biometris, Wageningen University & Research, Droevendaalsesteeg 1, 6706OB, Wageningen, The Netherlands

**Keywords:** Electronic health services, Medical records systems, Quality improvement, Health information exchange, Information and communication technologies

## Abstract

**Background:**

Care for people with an Intellectual Disability (ID) is complex: multiple health care professionals are involved and use different Health Information Systems (HISs) to store medical and daily care information on the same individuals. The objective of this study is to identify the HISs needs of professionals in ID care by addressing the obstacles and challenges they meet in their current HISs.

**Methods:**

We distributed an online questionnaire amongst Dutch ID care professionals via different professional associations and care providers. 328 respondents answered questions on their HISs. An inventory was made of HIS usage purposes, problems, satisfaction and desired features, with and without stratification on type of HIS and care professional.

**Results:**

Typical in ID care, two types of HISs are being used that differ with respect to their features and users: Electronic Client Dossiers (ECDs) and Electronic Patient Dossiers (EPDs). In total, the respondents mentioned 52 unique HISs. Groups of care professionals differed in their satisfaction with ECDs only. Both HIS types present users with difficulties related to the specifics of care for people with an ID. Particularly the much needed communication between the many unique HISs was reported a major issue which implies major issues with inter-operability. Other problems seem design-related as well.

**Conclusion:**

This study can be used to improve current HISs and design new HISs that take ID care professionals requirements into account.

**Supplementary Information:**

The online version contains supplementary material available at 10.1186/s12913-021-07256-9.

## Background

Around 0.85% of the Dutch population has an intellectual disability (ID) according to the definition of having an IQ below 70 [1]. People with an ID often live in long­term care facilities and have a large share in health care use in the Netherlands [2]. Care for people with an ID is complex and involves many professional caregivers. For example, a person with an ID is in close contact with a personal care aide who helps him or her with their daily activities and medication, but also sees a dentist, a general practitioner, and a physical therapist. All caregivers use some type of health information system (HIS) to register their activities and to ensure reimbursement of care provided. This is not without consequence: according to Hanekamp et al. [3], care professionals in long term care in the Netherlands spend 35% of their time on administrative tasks, mainly in their respective HISs.

Typically, HISs are not only used for reimbursement but also to assist professionals and organizations to contribute to high­quality, efficient patient care [4–6]. Well­functioning HISs, are therefore an important building block in the care for people with an ID [7, 8]. In the context of our research HISs contain and manage (medical) information about the person with the ID. Other frequently used terms for HISs in ID care are: electronic health records, and electronic client (or patient) dossiers/records.

Because of the role of HISs in, amongst other, reimbursement [9], patient safety [10], communication amongst professionals [11] and the time spent on administrative tasks in HISs [12], it is essential to know about the HISs used in the complex care for people with ID. It is, however, unknown which HISs are used in ID care, how these systems cater to the needs of different groups of professionals, and what features are used and needed. Reports from multiple countries suggested that different HISs are not interoperable [13–15]. Despite several studies on HISs in other forms of care, such as hospital care and GP care [16–20], little research has been done on the problems professional involved in ID care experience with HISs, and on their requirements for HISs. Software developers and researchers need to know about tasks, problems and requirements of care professionals to develop better systems. Therefore, the objective of this study is to identify for ID care, the needs of care professionals by taking inventory of their experiences and wishes for HISs. To fulfill the objective, we set up the following research question: *In care for people with ID, what are the key care professionals’ experiences with, and desired requirements for, HISs?* With the following sub­questions:

- Which HISs do care professionals for people with an ID use and for which purposes?
- How do care professionals for people with an ID assess their current HIS?
- Which features are care professionals for people with an ID missing in their current HISs?
- To answer these questions, we performed a cross­sectional study in which Dutch care professionals working in ID care received a questionnaire informed by semi­structured interviews.

## Methods

In the following, we describe the design of the online questionnaire, the recruitment of study participants, and data analysis.

### Questionnaire construction

As preparation of questionnaire design and to assure thorough familiarity with this diverse care field, we interviewed from our personal and professional networks fourteen health care professionals who differed in terms of their profession but who all worked in ID care. The semi­structured interviews lasted about an hour on average and during site visits, interviewees also showed us their local HISs. All interviewees agreed with audio­recording. Transcriptions of the recordings were analyzed to identify routing requirements and in vivo coded (using Atlas.ti 8) to identify possible answer categories for the standardized questionnaire.

From the interviews and site­visits we learned that two types of HISs were distinguished in practice which differed with respect to users and aim: Electronic Client Dossiers (ECDs) and Electronic Patient Dossiers (EPDs). Care professionals in ID care mainly used the ECDs to register daily activities for care continuity. The EPDs were used for clinical purposes by, for example, dentists and ID physicians. We, therefore, built three routings in the questionnaire: one for the ECDs, one for EPDs, and one for care professionals who used both kinds of systems.

The in vivo coding of the transcripts yielded a total of 18 features and 9 problems with HISs in ID care. Because of interview limitations, we also included an ‘Other, …. ’ answer option. The first concept of the questionnaire was pre­tested by means of a cognitive interview [21]. Based on this interview the questionnaire was improved, until after the third cycle no new issues appeared.

### Recruitment of study participants

We recruited a wide range of health care professionals working in ID care as study participants via various Dutch professional associations and care providers. We were unable to obtain e-mail lists of the members of the professional organizations and the employees of the institutions due to Dutch privacy regulations. Therefore, we used tailored recruitment methods. Personal care aides, nurses, resident assistants (for assisted living), and sheltered workshop guides/job coaches received an e­mail from long­term care providers and their professional association ‘Professional Association of Social Work Professionals’(BPSW). ID physicians were recruited by an e­mail from the ‘Dutch Association of ID­ physicians’(NVAVG). Remedial educationalists and behavioral therapists were recruited via an e­mail and the newsletter from their professional association ‘Dutch Association for Remedial Educationalists and Behavioral Therapists’(NVO). Psychologists were recruited via a LinkedIn post from the ‘Dutch Association for Healthcare Psychologists’(NVGzP). The ‘Dutch Association for Psychiatry’(NVvP) published an invitation for participation on their intranet. Pharmacists were approached by e­mail through the ‘Dutch Association of Pharmacists for People with an Intellectual Disability’(NVAPVG). Dentists were approached via the website, newsletter, and Facebook page of The ‘Central Consultation for Special Dentistry’, and the newsletter and Facebook page of ‘Association for the Promotion of Dental Care for the Disabled’(VBTGG). The ‘Dutch Association of Physical Therapists for People with an ID’(NVFVG) invited their members via their newsletter.

### Data collection procedure

After opening the online questionnaire, participants were requested to read information on the study’s objective and give informed consent. The first question inquired after profession and HIS(s) used to determine routing. The questionnaire continued with questions on satisfaction, features, and problems related to the HIS(s) and concluded with questions on gender, the region of employment, and work experience. Data were collected using Qualtrics [22]. Data collection took place between September 2nd 2019, and October 15th, 2019. All questionnaires are available upon request from the corresponding author. All methods were carried out in accordance with relevant guidelines and regulations.

### Data analysis

‘Other, ...’ answers were carefully read, and categorized by three researchers independently. Differences in classifications were discussed until consensus was reached. Then, new categories were added to the data file. We distinguished four groups of care professionals based on where they provide care, frequency (daily or not) and kind of professional training (academic or not): Daily care, Intellectual Disability physician, Mental Health and Development (MH&D), and Other care (see Table 1). Members of the Daily care group, although diverse in terms of profession, all work for a ID care provider. In the ID physician group, we also included ID physicians in training. The Mental Health and Development group works with people with ID on a daily basis, either within an ID healthcare provider or not. The remaining Other group is involved in care for, amongst others, people with an ID.

**Table 1:** The respondents per group of care professionals. Respondents were able to select one, or multiple professions

**Daily care**	
Personal care aide	85
Nurse	45
Resident assistant at assisted living	32
Sheltered workshop guide, job coach	13
Other^a^	9
Total	144
**ID physician**	
ID physician	83
ID physician resident	8
Total	91
**Mental health & Development**	
Remedial educationalist	22
Psychologist	19
Behavioural therapist	5
Psychiatrist	4
Therapist, other	3
Other^b^	3
Total	54
**Other care**	
Pharmacist	15
Dentist	9
Physical therapist	5
Medical Doctor	5
Other^c^	5
Total	39
**Total**	328

^a^: Service coordinator, Functional manager, Policy officer, Extramural care professional, Trainer, care coordinator, Case manager, ECD expert, and Maternity aide

^b^: Mental healthcare worker, Remedial teacher, and Prevention assistant

^c^: Dental laboratory technician, researcher, and three General practitioners

Respondents’ use and satisfaction with the ECDs and EPDs features (*N* = Respondents, Likert scale; 1 = very dissatisfied, 2 = dissatisfied, 3 = neither satisfied nor dissatisfied, 4 = satisfied, 5 = very satisfied)

We investigated differences between these groups, and between EPDs and ECDs. With the non­parametric Kruskal­Wallis test we tested (with *α* = 0*.*05) differences between the four groups on HIS’ satisfaction. All statistics were obtained with R.

## Results

### Descriptives of respondents

Of the 328 respondents in Table 1, 275 also answered the last question (i.e. 16.2% drop­out). All respondents answered three or more questions. Nearly half of the respondents belonged to the Daily care group (*n* = 144).

The respondents worked between one and 43 years in ID care, the median being 13 years (Table 2). Daily care professionals worked the longest in ID care with a median of 17.5 years. 80% of the respondents were women, only the ‘Other care’ group had a lower percentage, with 58%.

**Table 2:** Descriptive statistics on the respondents and systems

		Group				
		Daily Care	ID Physician	MH&D	Other Care	Total^a^
Years worked in ID care	N	120	82	38	30	270
	Median	17.5	10	11.5	10	13
	Range	1.5–43	2–43	1–35	2–39	1–43
Number of systems mentioned	N	143	139	54	39	375^b^
Number of unique systems	ECD	12	8	13	4	20
	EPD	0	12	8	19	32
%Women	N	122	83	39	31	275
	Percentage	83	82	82	58	80

^a^The number of respondents from this table differs from Table 1 due to missings

^b^Some care professionals use two systems

Which HISs do care professionals for people with an ID use and for which purposes?

In total, 52 unique HISs were identified (Table 2), of which the majority (32) were EPDs. Table 3 shows the features used in ECDs (*n* = 225) and EPDs (*n* = 118), and satisfaction/dissatisfaction with that feature. Overall, Patient/Client administration was most frequently mentioned, followed by Reporting and Client treatment and Support registration. The majority of ECD users indicated other registrations, e.g. freedom of movement, and visitations. In the Appendix in additional Table 1 results were shown for each of the four different groups of care professionals.

**Table 3:** Respondents’ use and satisfaction with the ECDs and EPDs features (*N* = Respondents, Likert scale; 1 = very dissatisfied, 2 = dissatisfied, 3 = neither satisfied nor dissatisfied, 4 = satisfied, 5 = very satisfied)

Feature	Users N	N	ECD Mode	Range	N	EPD Mode	Range
Patient/Client administration	238	149	4	1–5	89	4	2–5
Reporting	207	194	4	1–5	13^b^	5	1–5
Client treatment and support registration	194	183	4	1–5	11	4	2–5
Storage and document management	171	162	4	1–5	9^c^	4	2–4
Register medical patient information	120	10^a^	2	1–5	110	4	1–5
Financial administration and reimbursement	109	69	4	1–5	40	4	1–5
Calendar Management	101	36	4	1–4	65	4	1–5
Registration of consultation following structure	96	–	–	–	96	4	1–5
Making of letters	92	–	–	–	92	4	1–5
Test results from specialist/lab	90	–	–	–	90	4	1–5
Registration of diagnoses	81	–	–	–	81	3	1–5
Communication between team members	72	67	4	1–5	5^a^	5	2–5
Prescribe medication	70	–	–	–	70	3	1–5
Client portal	68	67	3	2–5	1^a^	3	–
Electronic exchange of patient/client dossier	62	–	–	–	62	2	1–5
Medication overview	15	–	–	–	15	4	3–5
Medication surveillance	14	–	–	–	14^c^	4	3–5
Other registrations	172	169	4	1–5	3^a^	NU^d^	3–5

^a^Obtained from ‘Other, …’

^b^Answer option provided to Dentists only

^c^Answer option provided to Pharmacists only

^d^*NU* Not Unique

### How do care professionals for people with an ID assess their current HIS?

The groups differed on their satisfaction level with respect to their ECD in general (Kruskal­ Wallis, *p* < 0.0001), with the Daily care group expressing higher levels of satisfaction than the other groups, in particular the Mental Health & Development group (Table 4). No evidence was found for different satisfaction levels with the general use of EPDs (Kruskal­Wallis, *p* = 0.061). For general satisfaction with their EPD, all groups’ modes were ‘Satisfied’.

**Table 4:** The care professionals’ satisfaction with their system per group. Their frequency (N), Mode, and range of satisfaction (Likert scale, 1 = very dissatisfied, 2 = dissatisfied, 3 = neither satisfied nor dissatisfied, 4 = satisfied, 5 = very satisfied)

	System		Group				
			Daily Care	ID Physician	MH&D	Other Care	Total
How satisfied or dissatisfied are you with your HIS in general?	ECD	N	143	63	40	4	250
		Mode	4	3	2	NU^a^	4
		Range	1–5	1–4	1–4	2–5	1–5
	EPD	N	–	77	14	36	127
		Mode	–	4	4	4	4
		Range	–	1–5	1–4	1–4	1–5
How satisfied or dissatisfied are you with the suitability of the HIS for ID care?	ECD	N	142	63	40	4	249
		Mode	4	3	3	3	4
		Range	2–5	1–5	1–5	3–5	1–5
	EPD	N	–	77	14	36	127
		Mode	–	2	2	3	3
		Range	–	2–5	1–4	2–5	1–5

^a^*NU* Not Unique

For the satisfaction with the suitability of ECDs for ID care a significant difference between groups was found (Kruskal­Wallis, *p* < 0.0001). Daily care professional’s reports ranged from dissatisfied to very satisfied, with the most frequently occurring judgment ‘satisfied’ (Table Table 4). Based on range and mode, the ID physicians and Mental Health & Development groupappear less satisfied with the suitability of the ECD for ID care. For EPDs, no evidence for differences between groups was found (Kruskal­Wallis, *p* = 0.12). The satisfaction with the suitable of EPDs for ID care appears rather low across all groups with the modes ‘dissatisfied’, and ‘neither satisfied nor dissatisfied’.

When we go back to Table 3 for more detailed information on dis/satisfaction with features, we see that although the modes suggested satisfaction with the most frequently used features of ECDs, there was no consensus: satisfaction ranged from very dissatisfied to very satisfied. The satisfaction with the less frequently mentioned ECD features, suggested that some people have experienced serious problems with the registration of medical patient information.

A similar situation can be seen for EPD features: although modes suggest that users were rather satisfied with most features, there was no consensus at all. The modes ‘neither satisfied nor dissatisfied’ for the features ‘registration of diagnoses’ and ‘prescribe medication’ should be seen as an important signal: not only because these features are crucial to medical treatment and monitoring but also because they support medication safety. The lowest mode, dissatisfaction, was found for the electronic exchange of patient/client dossier.

The overall most frequently mentioned problems related to HIS design were ‘hard to retrieve information from the system’ (185 times), followed by ‘difficult to exchange electronic client/patient records with other caregivers’ (Table 5). Most other problems mentioned by over 100 respondents related to system reliability: slow, and unavailable systems, and updates that changed the system.

**Table 5:** Most frequently identified HIS problems, for ECDs (*N* = 221 respondents) and EPDs (*N* = 111 respondents)

Problem	ECD	EPD	Total
Hard to retrieve information in system	124	61	185
Difficult to exchange electronic client/patient records with other caregiver	76	73	149
System is slow	103	44	147
Having to work in multiple systems at the same time	65	70	135
System is unavailable	86	40	126
Updates change the system	80	26	106
Hard to exchange information with other systems within care institution	74	8	82
Primary care classification method not differentiated enough for ID care	-^b^	75	75
Bad user interface	5	1	6
User roles and permissions	3	0	3
Other problems^a^	4	1	5
No problems at all	10	5	15

^a^Problems that could not be classified into one of the above problems

^b^Not provided as an answer option in the survey for this system

Based on the ‘Other … ’ answers we added two new problem categories: Bad user interface, and User roles and permissions. Only 15 of the 277 care professionals indicated they had experienced no problems with one of their systems. (Table 5). See additional Table 2 in the Appendix for data on group level.

Which features are care professionals for people with an ID missing in their current HISs? Eighty-eight respondents suggested desired, missing, features. We categorized these into 24 missing features (Table 6). The most frequently mentioned missing feature was the link with other systems (20 times), followed by features to provide an overview of data in the system and to prescribe and monitor medication (12 times). Features that were reported missing by one respondent only were: assign tasks, calendar management, financial management, taking history and making care­plan, open multiple dossiers at the same time, privacy robust procedures, suitability of system across disciplines, synchronization Windows and ECD, and ‘other registrations’. Additional Table 3 in the Appendix shows the missed features per group of care professionals.

**Table 6:** The features the care professionals reported missing. ECDs *N* = 51, EPDs *N* = 37

Missing Feature	ECD	EPD	Total
Link with other systems	8	12	20
Providing overview	6	6	12
Prescription management and monitoring	5	7	12
Information exchange	4	6	10
No feature but problem	8	1	9
Uploading of files	3	5	8
External correspondence	4	3	7
Clinical notes management	1	5	6
Electronic prescription	2	4	6
Access to parts of system	2	2	4
Epilepsy module	3	1	4
Detailed reporting	1	2	3
Lab information	1	2	3
Data search and filter	0	2	2
Insult registration	0	2	2
Other	6	4	10

## Discussion

### Reflection on the results

To the best of our knowledge, this is the first study on HISs in ID care that included both medical and non­medical care professionals who all report on well­being and medical issues concerning the same individuals. We identified ECDs used by all groups involved in residential care and EPDs for medical care. Over 300 respondents reported a total of 52 unique HISs. Care professionals used different HISs for a variety of tasks ranging from administration to treatment registration and reporting. They also reported support registration and a client portal, features specific to ID care and illustrating complexity of this care setting. Information is scattered over different systems which challenges professionals in ID care.

Often people with ID express medical problems, like pain, in behavioral change [23]. This implies that medical staff may experience difficulties because they can’t access the ECD and professionals in daily care may be able to do a better job if they have access to EPDs. Despite a reasonable overall satisfaction with HISs, nearly all respondents reported problems. Many problems related to HIS design (e.g. problems with the user interface), and reliability (slow, and outdated systems). Other problems stem from the organization of ID care, with multiple HISs, information exchange needs, and missing features.

### Related work

Previous studies reviewed HIS satisfaction and adoption level for one user group, i.e. medical doctors or nurses in hospitals [16–18, 24–26] or in other care domains [19, 20, 27–31]. In general, these studies showed dissatisfaction with HISs, despite high adoption rates. Comparability between these studies depends on the national organization of healthcare, and is therefore limited (e.g. the GP as gatekeeper for specialized care, the presence of an ID­physician) [32, 33]. Comparability with our study is also limited because the care for people with ID is characterized by a combination of support and care services [34–36].

Other studies [11, 37–41] assessed the adoption and satisfaction with HISs based on features but not on what was missing. Generally speaking these studies found, just like ours, similar levels of satisfaction with the features.

A study in the ID care setting, considered governmental databases as information systems is Karimi et al. [42]. The features they distinguished are, therefore, types of data saved in these databases, and resemble outcomes of our features ‘patient/client administration’, ‘reporting’, and ‘financial administration and reimbursement’. In their discussion, Karimi et al. [42] stressed the necessity of data exchange, which was also mentioned by our respondents.

Like ID care, geriatric care is a form of long­term care, but with better studies on HISs [43–45]. Wang and Biedermann [44] and Cherry et al. [45] primarily studied adoption and use of HISs, but not so much satisfaction. Sockolow et al. [43], conducted a survey on one HIS used in a geriatric community setting and concluded that although Philadelphian clinicians did not use the system as intended by the developers, they were satisfied with their HISs. We did not assess whether the care professionals used the systems as intended, since we did not study one HIS, but as it turned out, 52 HISs.

### Strengths & weaknesses

The collaboration with nine professionals’ associations and various institutions’ willingness to participate shows the importance of this study. With their help, we could review a wide range of HISs used in ID care. Despite the limited role of GPs in care for institutionalized people with an ID we have attempted to gain the interest of multiple professional organizations for GPs, unfortunately they did not want to participate. Nonetheless we were able to review the most common GP HISs, mainly thanks to the Dutch ID physicians. We believe our sample reflects the main professions responsible for the daily care of people with intellectual disabilities. Nonetheless, there are professions from which no representatives were included in our sample or which were under-represented such as speech therapists, nutritionists and medical doctors other than ID physicians. However, problems with information exchange between these professionals and the respondents of our survey could be reported and, indeed, have been mentioned.

From the interviews, we derived our online questionnaire, including the set of features, which we tested and enhanced with cognitive interviews. We chose not to use a standardized set of features and criteria [46, 47], since these appeared too limited, and too coarse for ID care. The questionnaire contained some questions with a fill­in ‘Other, … ‘answer option. By discussing and synthesizing these answers, we experienced the added value of authors from different disciplines.

For the satisfaction questions, we adopted Likert-type scales despite their metric limitations, because they are familiar and therefor easy for respondents. Despite our carefully designed questionnaire, the drop­out rate was 16%. Fortunately our data was rich, because many and various respondents gave ‘Other, ...’ answers.

### Practical implications

This study showed that the current EPDs are not satisfactory for the special needs in ID care. This indicates that in­depth knowledge of the specific ID care setting is necessary for the development of HISs, since it is more complex than other types of care. In the Netherlands, complexity might further increase due to the recently introduced legal obligation to give people with ID access to their own dossier [48]. This access is usually provided through a client portal which is also listed as a feature in Table 3. In practice, relatives often also have access to this system, which allows them to monitor care. Also, the legal representatives make the decisions about what happens to the data of the people with intellectual disabilities, if they cannot decide about these topics this themselves. This creates a complexity for system access and usage of the data. Furthermore, the large number of identified HISs (52) causes multiple interoperability problems.

This complexity, and the aforementioned problems with usability calls for a modular system, that serves as an ECD, EPD, and client portal, where ID modules with particular features can be removed and added as required. The modular system may reduce costs, because of it’s decomposability.

A modular HIS for ID care should be based on standards for information exchange and a reference software architecture [49]. Furthermore, a reference architecture may help make data in ID care more suitable for research by combining privacy standards with the FAIR data principles [35].

## Conclusions

In conclusion, answering the general research question, in care for people with ID a wide range of different HISs is used. Despite some satisfaction with the HISs, many problems remain to be solved. Future work will be the design of a reference architecture for modular HISs in ID Care.

## Supplementary Information

**Additional file 1. **Table 1. Respondents’ satisfaction with the features they reported using (*N* = Respondents who use the feature, followed by Mode and Range; 1 = very dissatisfied, 2 = dissatisfied, 3 = neither satisfied nor dissatisfied, 4 = satisfied, 5 = very satisfied). Table 2. (Table 5 by care professional groups) Most frequently identified HIS problems, for each group of care professionals. Daily care = 123 responses, Intellectual Disability Physician = 133 responses, Mental Health and Development = 44 responses, Other care = 32 responses. Table 3. (Table 6 by care professional groups): The features the care professionals reported missing. Daily care = 14 responses, Intellectual Disability Physician = 48 responses, Mental Health and Development = 12 responses, Other care = 10 responses.

## Data Availability

The individual surveys analysed during the current study are not publicly available due to confidentiality. Aggregated results are available from the corresponding author on reasonable request.

## Abbreviations

HIS: Health Information System
ID: Intellectual Disability
ECD: Electronic Client Dossier
EPD: Electronic Patient Dossier
MH&D: Mental Health and Development
