# Supplementary material for: State of the practice of health information systems: a survey study amongst health care professionals in intellectual disability care
Source: BMC Health Serv Res. 2021 Nov 18;21:1247. doi: 10.1186/s12913-021-07256-9 (PMC8603513; doi:10.1186/s12913-021-07256-9)
Supplement: Supplementary file 1 — Additional file 1. Table 1. Respondents’ satisfaction with the features they reported using (N = Respondents who use the feature, followed by Mode and Range; 1 = very dissatisfied, 2 = dissatisfied, 3 = neither satisfied nor dissatisfied, 4 = satisfied, 5 = very satisfied). Table 2. (Table 5 by care professional groups) Most frequently identified HIS problems, for each group of care professionals. Daily care = 123 responses, Intellectual Disability Physician = 133 responses, Mental Health and Development = 44 responses, Other care = 32 responses. Table 3. (Table 6 by care professional groups): The features the care professionals reported missing. Daily care = 14 responses, Intellectual Disability Physician = 48 responses, Mental Health and Development = 12 responses, Other care = 10 responses. [file 12913_2021_7256_MOESM1_ESM.zip › 2021-08-17-BMC-survey-Appendix.docx]

Appendix

August 17, 2021

Appendices

1

Additional table 1: (Table 3 by care professional groups): Respondents’ satisfaction with the features they reported using (N = Respondents who use the feature, followed by Mode and Range; 1 = very dissatisfied, 2 = dissatisfied, 3= neither satisfied nor dissatisfied, 4 = satisfied, 5 = very satisfied)

| **Feature** | **Users**  N | N | **Daily ca**  Mode | **re**  Range | **Intellectual Disability Physician**  N Mode Range | | | **Mental Health and**  N Mode | | | **Development**  Range | **Other ca**  Mode | **re**  Range |
| --- | --- | --- | --- | --- | --- | --- | --- | --- | --- | --- | --- | --- | --- |
| Patient/Client administration | 238 | 94 | 4 | 1-5 | 84 | 4 | 1-5 | 36 | 4 | 1-5 | 24 | 4 | 2-5 |
| Reporting | 207 | 121 | 4 | 1-5 | 37 | 4 | 1-5 | 33 | 3 | 1-5 | 16c | 5 | 1-5 |
| Client treatment and support registration | 194 | 108 | 4 | 1-5 | 38 | 3 | 1-5 | 35 | 2 | 1-5 | 13 | 4 | 2-5 |
| Storage and document management | 171 | 98 | 4 | 1-5 | 27 | 4 | 1-5 | 34 | 4 | 1-5 | 12d | 4 | 2-5 |

N

Register medical patient information 120 2a

| NUb | 2-5 | 80 | 4 | 1-5 |  | 9 | 3 | 1-4 |  | 29 | 4 | 1-5 |
| --- | --- | --- | --- | --- | --- | --- | --- | --- | --- | --- | --- | --- |

| Financial administration and reimbursement | 109 | 27 | 4 | 2-5 | 33 | 3 | 1-5 | 25 | 4 | 1-4 | 24 | 4 | 2-5 |
| --- | --- | --- | --- | --- | --- | --- | --- | --- | --- | --- | --- | --- | --- |
| Calendar Management | 101 | 19 | 4 | 1-4 | 44 | 4 | 1-5 | 21 | 3 | 1-4 | 17 | 4 | 2-5 |
| Registration of consultation following structure | 96 | - | - | - | 73 | 4 | 2-3 | 10 | 4 | 2-4 | 13 | 4 | 1-5 |
| Making of letters | 92 | - | - | - | 69 | 4 | 1-5 | 9 | 3 | 1-4 | 14 | 4 | 3-5 |
| Test results from specialist/lab | 90 | - | - | - | 73 | 4 | 1-5 | 7 | 3 | 1-4 | 10 | 3 | 3-5 |
| Registration of diagnoses | 81 | - | - | - | 68 | 3 | 1-5 | 11 | 4 | 1-4 | 2 | NUb | 4-5 |

3a NUb 2-5

| Prescribe medication | 70 | - | - | - | 48 | 3 | 1-5 | - | - | - | 22 | 4 | 1-5 |
| --- | --- | --- | --- | --- | --- | --- | --- | --- | --- | --- | --- | --- | --- |
| Client portal | 68 | 42 | 4 | 2-4 | 15 | 3 | 2-5 | 11 | 3 | 2-5 | - | - | - |
| Electronic exchange of patient/client dossier | 62 | - | - | - | 45 | 3 | 2-5 | 5 | 2 | 1-5 | 12 | 5 | 2-5 |

| Communication between team members | 72 | 35 | 4 | 1-5 | 22 | 4 | 1-5 | 12 | 4 | 1-4 |
| --- | --- | --- | --- | --- | --- | --- | --- | --- | --- | --- |

Medication overview

| Medication surveillance | 14 | - | - | - | - | - | - | - | - | - |
| --- | --- | --- | --- | --- | --- | --- | --- | --- | --- | --- |
| Other registrations | 172 | 98 | 4 | 1-5 | 39 | 4 | 1-5 | 30 | 3 | 1-5 |

15 - - -

1a 3 -

- - -

14d 4 4-5

14 4 3-5

5a NUb 2-5

aObtained from “Other, ...”

bNU: Not Unique

cAnswer option provided to Dentists only

dAnswer option provided to Pharmacists only

Additional table 2: (Table 5 by care professional groups) Most frequently identified HIS problems, for each group of care

professionals. Daily care = 123 responses, Intellectual Disability Physician = 133 responses, Mental Health and Development = 44 responses, Other care = 32 responses

**Problem**

| Daily Care | Intellectual Disability Physician |  | Mental Health and Development | Other Care |  | Total |
| --- | --- | --- | --- | --- | --- | --- |
| 60 |  | 80 | 30 |  | 15 | 185 |
| 29 |  | 77 | 21 |  | 22 | 149 |
| 59 |  | 58 | 18 |  | 12 | 147 |
| 17 |  | 86 | 15 |  | 17 | 135 |
| 55 |  | 47 | 18 |  | 6 | 126 |
| 53 |  | 29 | 17 |  | 7 | 106 |
| 36 |  | 24 | 12 |  | 10 | 82 |
| -b |  | 64 | 6 |  | 5 | 75 |
| 3 1 2 0  1 2 0 0  2 2 1 0  9 3 0 3 | | | | | | 6  3  5  15 |

Hard to retrieve information in system

Diﬀicult to exchange electronic client/patient dossiers with other caregiver

System is slow

Having to work in multiple systems at the same time System is unavailable

Updates change the system

Hard to exchange information with other systems within care institution

Primary care classification method not

| differentiated enough for ID care  Bad user interface |  | | | | |
| --- | --- | --- | --- | --- | --- |
| User roles and permissions |  |  |  |  |  |
| Other problemsa |  |  |  |  |  |
| No problems at all |  |  |  |  |  |

aProblems that could not be classified into one of the above problems

bNot provided as an answer option in the survey for this sy stem

Additional table 3:(Table 6 by care professional groups): The features the care professionals reported missing. Daily care = 14 responses, Intellectual Disability Physician = 48 responses, Mental Health and Development = 12 responses, Other care = 10 responses

| Missing Feature | Daily Care | Intellectual Disability  Physician |  | Mental Health and  Development | Other Care | Total |  |
| --- | --- | --- | --- | --- | --- | --- | --- |
| Link with other systems | 1 |  | 16 | 0 | 3 |  | 20 |
| Providing overview | 3 |  | 7 | 1 | 1 |  | 12 |
| Prescription management and monitoring | 1 |  | 8 | 0 | 3 |  | 12 |
| Information exchange | 1 |  | 7 | 1 | 1 |  | 10 |
| No feature but problem | 1 |  | 5 | 3 | 0 |  | 9 |
| Uploading of files | 3 |  | 3 | 1 | 1 |  | 8 |
| External correspondence | 0 |  | 5 | 2 | 0 |  | 7 |
| Clinical notes management | 0 |  | 5 | 0 | 1 |  | 6 |
| Electronic prescription | 0 |  | 5 | 0 | 1 |  | 6 |
| Access to parts of system | 0 |  | 4 | 0 | 0 |  | 4 |
| Epilepsy module | 1 |  | 2 | 0 | 1 |  | 4 |
| Detailed reporting | 1 |  | 2 | 0 | 0 |  | 3 |
| Lab information | 0 |  | 2 | 0 | 1 |  | 3 |
| Data search and filter | 0 |  | 1 | 0 | 1 |  | 2 |
| Insult registration | 0 |  | 2 | 0 | 0 |  | 2 |
| Other | 2 |  | 2 | 5 | 1 |  | 10 |
