# Supplementary material for: State of the practice of health information systems: a survey study amongst health care professionals in intellectual disability care
Source: BMC Health Serv Res. 2021 Nov 18;21:1247. doi: 10.1186/s12913-021-07256-9 (PMC8603513; doi:10.1186/s12913-021-07256-9)
Supplement: Supplementary file 1 — Additional file 1. Table 1. Respondents’ satisfaction with the features they reported using (N = Respondents who use the feature, followed by Mode and Range; 1 = very dissatisfied, 2 = dissatisfied, 3 = neither satisfied nor dissatisfied, 4 = satisfied, 5 = very satisfied). Table 2. (Table 5 by care professional groups) Most frequently identified HIS problems, for each group of care professionals. Daily care = 123 responses, Intellectual Disability Physician = 133 responses, Mental Health and Development = 44 responses, Other care = 32 responses. Table 3. (Table 6 by care professional groups): The features the care professionals reported missing. Daily care = 14 responses, Intellectual Disability Physician = 48 responses, Mental Health and Development = 12 responses, Other care = 10 responses. [file 12913_2021_7256_MOESM1_ESM.zip › 2021-08-17-BMC-survey-Appendix.pdf]

Appendix

August 17, 2021

Appendices

Additional table 1: (Table 3 by care professional groups): Respondents' satisfaction with the features they reported using (N = Respondents who use the feature, followed by Mode and Range; 1 = very dissatisfied, 2 = dissatisfied, 3= neither satisfied nor dissatisfied, 4 = satisfied, 5 = very satisfied)

	Users	Daily care			Intellectual Disability Physician			Mental Health and Development			Other care		
**Feature**	N	N	Mode	Range	N	Mode	Range	N	Mode	Range	N	Mode	Range
Patient/Client administration	238	94	4	1-5	84	4	1-5	36	4	1-5	24	4	2-5
Reporting	207	121	4	1-5	37	4	1-5	33	3	1-5	16[c]	5	1-5
Client treatment and support registration	194	108	4	1-5	38	3	1-5	35	2	1-5	13	4	2-5
Storage and document management	171	98	4	1-5	27	4	1-5	34	4	1-5	12[d]	4	2-5
Register medical patient information	120	2[a]	NU[b]	2-5	80	4	1-5	9	3	1-4	29	4	1-5
Financial administration and reimbursement	109	27	4	2-5	33	3	1-5	25	4	1-4	24	4	2-5
Calendar Management	101	19	4	1-4	44	4	1-5	21	3	1-4	17	4	2-5
Registration of consultation following structure	96	-	-	-	73	4	2-3	10	4	2-4	13	4	1-5
Making of letters	92	-	-	-	69	4	1-5	9	3	1-4	14	4	3-5
Test results from specialist/lab	90	-	-	-	73	4	1-5	7	3	1-4	10	3	3-5
Registration of diagnoses	81	-	-	-	68	3	1-5	11	4	1-4	2	NU[b]	4-5
Communication between team members	72	35	4	1-5	22	4	1-5	12	4	1-4	3[a]	NU[b]	2-5
Prescribe medication	70	-	-	-	48	3	1-5	-	-	-	22	4	1-5
Client portal	68	42	4	2-4	15	3	2-5	11	3	2-5	-	-	-
Electronic exchange of patient/client dossier	62	-	-	-	45	3	2-5	5	2	1-5	12	5	2-5
Medication overview	15	-	-	-	1[a]	3	-	-	-	-	14[d]	4	4-5
Medication surveillance	14	-	-	-	-	-	-	-	-	-	14	4	3-5
Other registrations	172	98	4	1-5	39	4	1-5	30	3	1-5	5[a]	NU[b]	2-5

[a]Obtained from "Other, ..."

[b]NU: Not Unique

[c]Answer option provided to Dentists only

[d]Answer option provided to Pharmacists only

Additional table 2: (Table 5 by care professional groups) Most frequently identified HIS problems, for each group of care professionals.

**Problem**	Daily Care	Intellectual Disability Physician	Mental Health and Development	Other Care	Total
Hard to retrieve information in system	60	80	30	15	185
Difficult to exchange electronic client/patient dossiers with other caregiver	29	77	21	22	149
System is slow	59	58	18	12	147
Having to work in multiple systems at the same time	17	86	15	17	135
System is unavailable	55	47	18	6	126
Updates change the system	53	29	17	7	106
Hard to exchange information with other systems within care institution	36	24	12	10	82
Primary care classification method not differentiated enough for ID care	-[b]	64	6	5	75
Bad user interface	3	1	2	0	6
User roles and permissions	1	2	0	0	3
Other problems[a]	2	2	1	0	5
No problems at all	9	3	0	3	15

[a]Problems that could not be classified into one of the above problems

[b]Not provided as an answer option in the survey for this system

Additional table 3:(Table 6 by care professional groups): The features the care professionals reported missing.

Missing Feature	Daily Care	Intellectual Disability Physician	Mental Health and Development	Other Care	Total
Link with other systems	1	16	0	3	20
Providing overview	3	7	1	1	12
Prescription management and monitoring	1	8	0	3	12
Information exchange	1	7	1	1	10
No feature but problem	1	5	3	0	9
Uploading of files	3	3	1	1	8
External correspondence	0	5	2	0	7
Clinical notes management	0	5	0	1	6
Electronic prescription	0	5	0	1	6
Access to parts of system	0	4	0	0	4
Epilepsy module	1	2	0	1	4
Detailed reporting	1	2	0	0	3
Lab information	0	2	0	1	3
Data search and filter	0	1	0	1	2
Insult registration	0	2	0	0	2
Other	2	2	5	1	10
